# Lipidomic profiling in Crohn's disease: Abnormalities in phosphatidylinositols, with preservation of ceramide, phosphatidylcholine and phosphatidylserine composition

**DOI:** 10.1016/j.biocel.2012.06.016

**Published:** 2012-11

**Authors:** Gavin W. Sewell, Yusuf A. Hannun, Xianlin Han, Grielof Koster, Jacek Bielawski, Victoria Goss, Philip J. Smith, Farooq Z. Rahman, Roser Vega, Stuart L. Bloom, Ann P. Walker, Anthony D. Postle, Anthony W. Segal

**Affiliations:** a Division of Medicine, UCL, 5 University Street, London, United Kingdom; b Department of Medicine, Stony Brook University, Stony Brook, NY, United States; c Department of Biochemistry and Molecular Biology, Medical University of South Carolina, Charleston, SC, United States; d Sanford-Burnham Medical Research Institute, Orlando, FL, United States; e Division of Infection, Inflammation & Immunity, University of Southampton, School of Medicine, Southampton General Hospital, Mailpoint 803, South Block, Tremona Road, Southampton, United Kingdom; f Department of Gastroenterology, University College Hospital, 235 Euston Road, London, United Kingdom

**Keywords:** CCT, Phosphocholine cytidylyltransferase, CD, Crohn's disease, GWAS, Genome-wide association study, HC, Healthycontrol, HkEc, Heat-killed *Escherichia coli*, PA, Phosphatidic acid, PC, Phosphatidylcholine, PI, Phosphatidylinositol, PS, Phosphatidylserine, TNF, Tumor necrosis factor, Crohn's disease, Macrophage, Lipids, Ceramide, Sphingolipid, Phospholipid

## Abstract

Crohn's disease is a chronic inflammatory condition largely affecting the terminal ileum and large bowel. A contributing cause is the failure of an adequate acute inflammatory response as a result of impaired secretion of pro-inflammatory cytokines by macrophages. This defective secretion arises from aberrant vesicle trafficking, misdirecting the cytokines to lysosomal degradation. Aberrant intestinal permeability is also well-established in Crohn's disease. Both the disordered vesicle trafficking and increased bowel permeability could result from abnormal lipid composition. We thus measured the sphingo- and phospholipid composition of macrophages, using mass spectrometry and stable isotope labelling approaches. Stimulation of macrophages with heat-killed *Escherichia coli* resulted in three main changes; a significant reduction in the amount of individual ceramide species, an altered composition of phosphatidylcholine, and an increased rate of phosphatidylcholine synthesis in macrophages. These changes were observed in macrophages from both healthy control individuals and patients with Crohn's disease. The only difference detected between control and Crohn's disease macrophages was a reduced proportion of newly-synthesised phosphatidylinositol 16:0/18:1 over a defined time period. Shotgun lipidomics analysis of macroscopically non-inflamed ileal biopsies showed a significant decrease in this same lipid species with overall preservation of sphingolipid, phospholipid and cholesterol composition.

## 1 Introduction

A number of physiological processes have been shown to be disturbed in Crohn's disease (CD). A recently described manifestation is the failure of acute inflammation, resulting in impaired clearance of bacteria from the tissues. This is due to mistargeting of pro-inflammatory mediators to lysosomal degradation in macrophages as a result of aberrant vesicle trafficking (Smith et al., 2009). Autophagy (Cooney et al., 2010) and apoptosis (Palmer et al., 2009) have also been shown to be abnormal in CD, and intestinal permeability is increased in this condition (Hollander et al., 1986).

Sphingolipids and phospholipids play key roles in the modulation of inflammation and immunity (El et al., 2006). Ceramide and ceramide-1-phosphate act to reduce tumor necrosis factor (TNF) release (Jozefowski et al., 2010), most likely via post-translational regulation of TNF and modulation of TNF converting enzyme activity (Rozenova et al., 2010). Furthermore, ceramides have important roles in the control of autophagy, a process strongly implicated in the pathogenesis of CD (Barrett et al., 2008). Addition of exogenous ceramide induces autophagy (Scarlatti et al., 2004), which may relate to effects on signalling networks or changes in the biophysical membrane properties (Zheng et al., 2006).

Phosphatidylcholine (PC) is an important structural component of all cell membranes, including intracellular vesicles (Howe and McMaster, 2001). In macrophages, the generation of PC may play a role in differentiation (Ecker et al., 2010) and pro-inflammatory cytokine release (Tian et al., 2008). Murine macrophages deficient in phosphocholine cytidylyltransferase (CCT-α) secrete reduced levels of TNF and IL-6 in response to lipopolysaccharide (LPS) stimulation, as a result of abnormal post-translational processing and retention of these molecules in the Golgi apparatus, similar to the situation observed in CD macrophages.

Phosphatidylinositol (PI) is an important structural phospholipid, and also a substrate for lipid kinases and phosphatases, which can generate phosphoinositide derivatives (PIPs). PIPs are critical second messenger molecules in pathways involved in the control of cytoskeletal re-organisation and membrane trafficking (Odorizzi et al., 2000). PI 3-kinase, an enzyme that converts PI species to phosphatidylinositol-3,4,5-trisphosphate, is important for TNF trafficking from the Golgi apparatus to the plasma membrane in macrophages (Low et al., 2010).

Various studies have suggested alterations in lipid metabolism in CD. Positive correlations exist between dietary fat consumption and the development of CD (Amre et al., 2007; Shoda et al., 1996). Genome-wide association studies (GWAS) have identified CD-associated variants in loci containing genes related to lipid metabolism. Specifically, a locus containing the *ORMDL3* gene confers susceptibility to CD (Barrett et al., 2008). *ORM* genes are involved in sphingolipid homeostasis; the ORM proteins encoded by these genes act as negative regulators of sphingolipid metabolism (Breslow et al., 2010). Furthermore, the recent GWAS meta-analysis identified a CD-associated SNP in close proximity to *FADS1* (Franke et al., 2010), which encodes the fatty acid desaturase 1 enzyme. Genetic variation in this gene is associated with alterations in the fatty acid composition in serum phospholipids (Schaeffer et al., 2006).

Studies conducted on biological samples have demonstrated decreased membrane fluidity in erythrocytes from CD patients, with concomitant increases in sphingomyelin and reductions in phosphatidylcholine and polyunsaturated acyl chains of phospholipid (Aozaki, 1989). Increased concentrations of lactosylceramide have been reported in bowel biopsies from CD patients (Stevens et al., 1988), although it is possible that these changes were secondary to inflammation. There is somewhat conflicting evidence describing fatty acid abnormalities in CD, including in plasma phospholipids (Esteve-Comas et al., 1992, 1993; Geerling et al., 1999) and PBMCs (Trebble et al., 2004). Lipids from adipose and lymphoid tissues contain more saturated but fewer polyunsaturated fatty acids, with preferential depletion of n-6 polyunsaturates in lymphoid tissue (Westcott et al., 2005).

Macrophage phospholipid and sphingolipid composition have not been previously investigated in CD. Given the possibility that macrophage sphingolipid or phospholipid composition could underlie the defective cytokine secretion from macrophages that is observed in CD, these molecules were quantified using high performance liquid chromatography tandem mass spectrometry (HPLC-MS). Phospholipid composition and dynamics were investigated using stable isotope labelling and electrospray ionisation mass spectrometry (ESI-MS).

## 2 Materials and methods

### 2.1 Subject recruitment and selection

Patients from University College London Hospitals Foundation NHS Trust fulfilled criteria for the diagnosis of CD (Lennard-Jones, 1989). All patients in the macrophage studies were between 18 and 75 years of age and had quiescent disease, as determined by the Harvey-Bradshaw disease activity index of <3 (Harvey and Bradshaw, 1980), and were receiving either no treatment or a stable dose (for the preceding 3 months) of 5-aminosalicylates (5-ASA) alone. Healthy volunteers were between 18 and 75 years of age and were not receiving immunosuppressant medication. Demographics of patients included in these studies are shown (Table 1).

For shotgun lipidomics investigations, ileal biopsies were obtained from CD patients (*n* = 5) and control individuals (*n* = 5). Two of the patients had macroscopic and microscopic evidence of active disease; the remaining patients had macroscopic and histologic features consistent with quiescent disease. Three patients were receiving no treatment, one was receiving 5-aminosalicylates and one was receiving methotrexate. Two patients had previous ileal resections; in these cases biopsies from the neoterminal ileum were obtained. In all cases biopsies were taken from macroscopically non-inflamed bowel.

Ethical approval was obtained from the Joint UCL/UCLH Committees on the Ethics of Human Research (project number 02/0324). No subject was studied more than once in each of the different sets of experiments.

### 2.2 Primary macrophage isolation, culture and stimulation

Peripheral blood mononuclear cells were isolated from venous blood samples as previously described (Smith et al., 2009). After 5 days of culture, cells were harvested, resuspended in X-Vivo-15 medium (Cambrex, MD, USA) and plated into BD Falcon™ culture plates.

### 2.3 Sphingolipid analysis of cultured macrophages

Monocyte-derived macrophages were stimulated for 4 h with heat-killed *Escherichia coli* (HkEc), as previously described (Smith et al., 2009). Cells were harvested in PBS, resuspended in 200 μl PBS and sonicated. 10 μl aliquots were obtained for protein determination.

The ceramide content of the solution remaining was determined using HPLC-MS by established methods (Bielawski et al., 2009). Samples were quantified by HPLC-MS on a Thermo Finnigan TSQ 7000 triple quadrupole mass spectrometer operating in a Multiple Reaction Monitoring positive ionisation mode. Sphingolipid concentrations were normalised in relation to total protein concentrations.

### 2.4 BCA assay

The protein content of samples was determined using the bicinchoninic acid (BCA) assay (Thermo Fisher Scientific Inc) with bovine serum albumin as standard.

### 2.5 Preparation of samples for phospholipid analysis and stable isotope incubation

After overnight incubation, medium was removed and replaced with X-vivo-15 (Cambrex) supplemented with deuterated choline (*methyl*-D_*9*_-choline, 100 μg/ml, Sigma Aldrich), deuterated inositol (*myo*-D_6_-inositol, 100 μg/ml, C/D/N isotopes, Quebec) and deuterated serine (serine-D_3_, 100 μg/ml C/D/N isotopes), in the presence or absence of HkEc (2.5:1).

Macrophages were incubated with the stable isotope-labelled compounds for 3 h at 37 °C in an atmosphere of 5% (v/v) CO_2_. Subsequently, medium was removed and cells were washed with Hanks Balanced Salt Solution (Invitrogen). The cells were lysed in 1 ml ice-cold methanol for lipid extractions.

### 2.6 Phospholipid extraction and analysis by electrospray ionisation mass spectrometry

Total lipid was extracted from macrophages using chloroform and methanol as described previously (Bligh and Dyer, 1959). Samples were reconstituted in 30 μl of a solution containing 20% butanol, 60% methanol, 16% water and 4% concentrated aqueous NH_3_ and introduced by direct infusion into a triple quadrupole mass spectrometer (Quattro Ultima, Micromass, UK) equipped with a nanoflow electrospray ionisation interface.

Phospholipid and neutral lipid species, both endogenous and with incorporated stable isotope-labelled substrates, were selectively detected and quantified from a variety of precursor (P) and neutral loss (NL) scans. Phosphatidylcholine (PC) was analysed in positive ionisation as P184+ and P193+ scans for endogenous and newly synthesised (D_9_) PC. Phosphatidylinositol (PI) and phosphatidylserine (PS) were analysed in negative ionisation, as P241- and P247- scans for endogenous PI and newly synthesised (D_6_) PI respectively, and NL87- and NL90- for endogenous PS and newly synthesised (D_3_) PS respectively. Data were processed using MassLynx software (Waters) and analysed using a custom-designed macro (Postle et al., 2011). Correction for the ^13^C isotope was performed prior to calculation of percentage composition and incorporation of labelled phospholipid head groups. The fractional incorporations of *methyl*-D_9_-choline, *myo*-D_6_-inositol and serine-D_3_ into PC, PI and PS species respectively were calculated relative to the total abundance. Only species of PC, PI and PS that constituted >2% of the total molar percentage of PC, PI or PS respectively were considered quantifiable.

### 2.7 Shotgun lipidomics analysis of ileal biopsies

Shotgun lipidomics analysis was performed as described previously (Han et al., 2004). Briefly, samples were homogenised in 1 ml ice-cold 50 mmol/l LiCl. Protein content was determined using the BCA assay. Internal standards, including dimyristoylphosphatidylcholine (15 nmol/mg protein), dimyristoylphosphatidylserine (1 nmol/mg protein), 1,2-dipentadecanoyl-*sn*-glycero-3-phosphoglycerol (4.2 nmol/mg protein), 1,2-dipentadecanoyl-*sn*-glycero-3-phosphoethanolamine (18.75 nmol/mg protein), 17C18 ceramide (40 pmol/mg protein) and triheptadecenoylglycerol (10 nmol/mg protein) were added and lipid extraction performed with a modified Bligh and Dyer procedure. ESI-MS was performed using a triple-quadrupole mass spectrometer (ThermoElectron TSQ Quantum Ultra, San Jose, CA, USA) (Han et al., 2004).

### 2.8 Statistical analysis

Statistical analysis was determined using a paired or unpaired student *t*-test in Microsoft Excel as appropriate. A *p*-value of *p* < 0.05 was considered statistically significant. For shotgun lipidomics experiments, a threshold *p*-value of *p* < 0.01 was used.

## 3 Results

### 3.1 Sphingolipid composition of macrophages is altered after stimulation with *E. coli* but does not differ between HC and CD macrophages

**Fig. 1:**
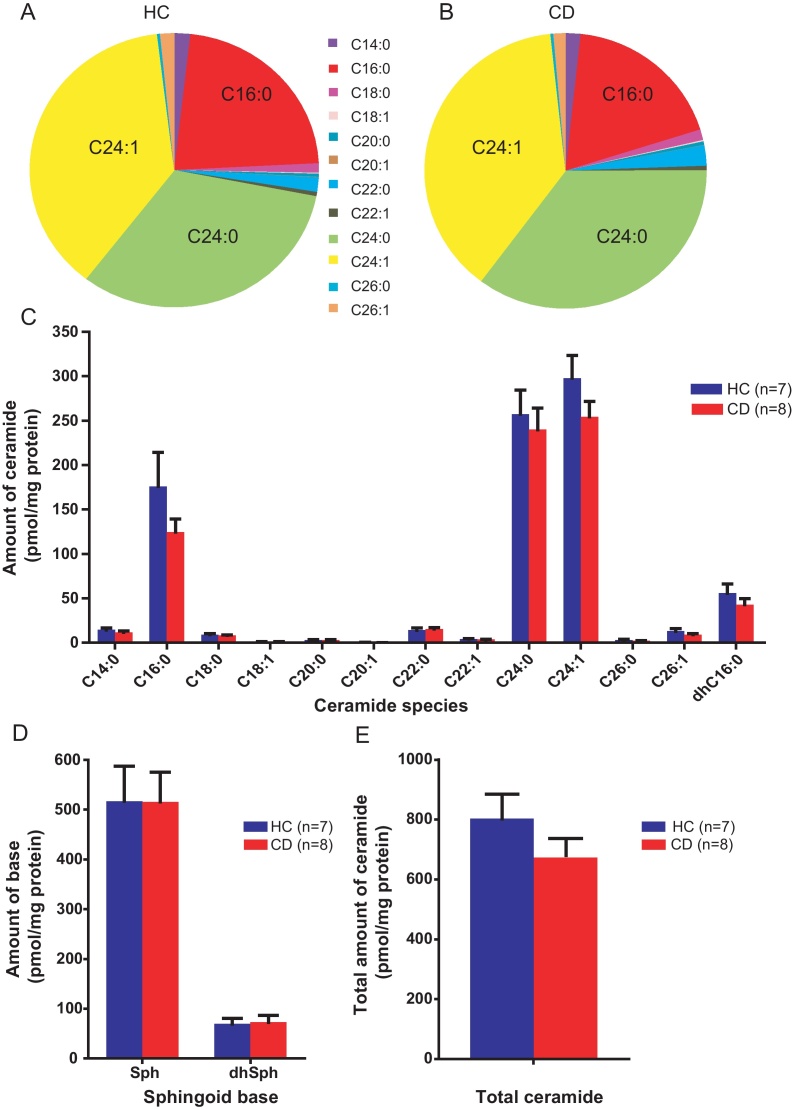
Ceramide and sphingoid base composition of CD macrophages is unaltered. The amounts of individual ceramide species were quantified in HC (*n* = 7) and CD (*n* = 8) macrophages by HPLC-MS. (A and B) Pie charts depicting the proportion of various ceramides in HC and CD macrophages respectively, expressed as a percentage of the total amount of ceramide detected. (C) The amounts of individual ceramide species (in pmol/mg protein) in unstimulated HC and CD macrophages. No significant differences were identified between HC and CD. (D) The amounts of sphingosine (Sph) and dihydrosphingosine (dhSph) in HC and CD macrophages. (E) Total amounts of ceramide in HC and CD macrophages. Results are expressed as mean + SEM.

The predominant ceramide species in both healthy control (HC) and CD macrophages were the C16:0, C24:0 and C24:1 ceramides (Fig. 1A and B). There were no significant differences in the mean amounts of any ceramide species, dihydroceramide or sphingoid base (Fig. 1C and D) or total ceramide (Fig. 1E) between HC (*n* = 7) and CD (*n* = 8) macrophages in the unstimulated state.

**Supplementary Fig. 1:**
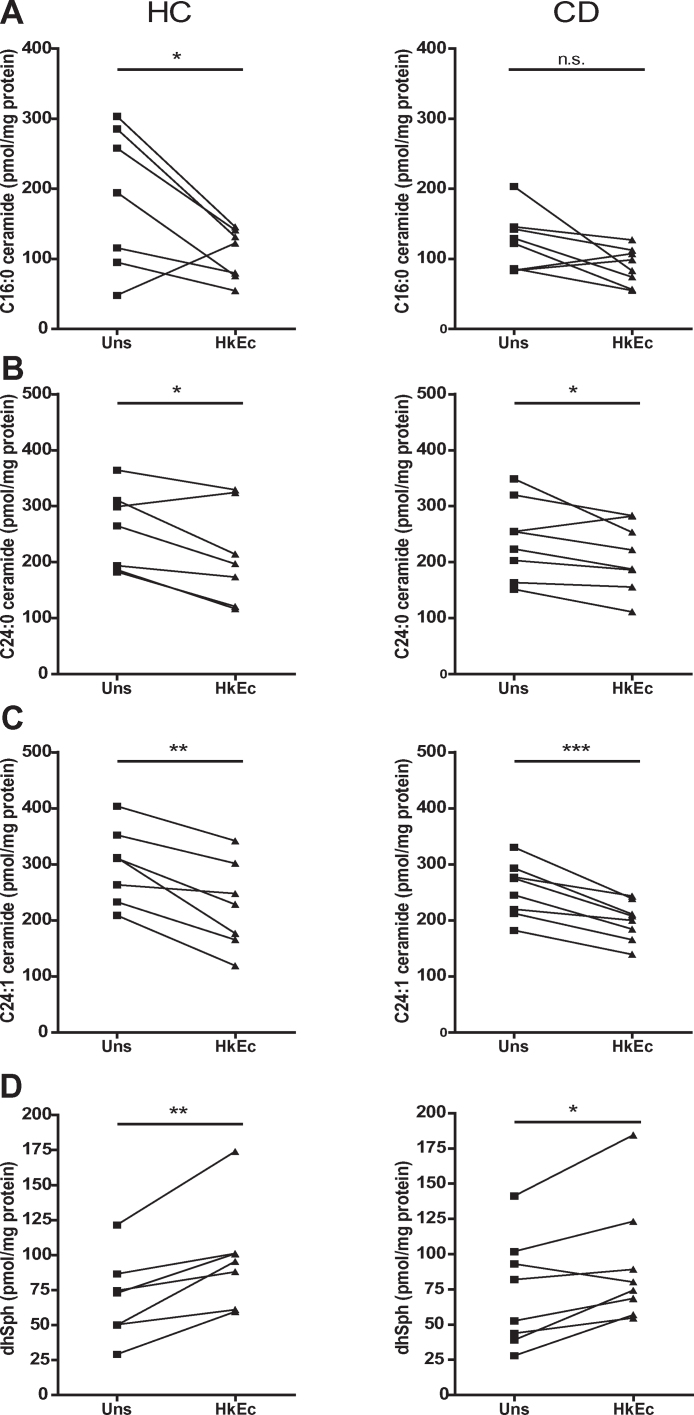
Ceramide and sphingoid bases in HC and CD macrophages after HkEc stimulation. Alterations in the amount of (A) C16:0, (B) C24:0, (C) C24:1 ceramides and (D) dihydrosphingosine (dhSph) are shown for HC (left panel) and CD (right panel) macrophages. * represents *p* < 0.05, ** *p* < 0.01 and *** *p* < 0.001.

**Supplementary Fig. 2:**
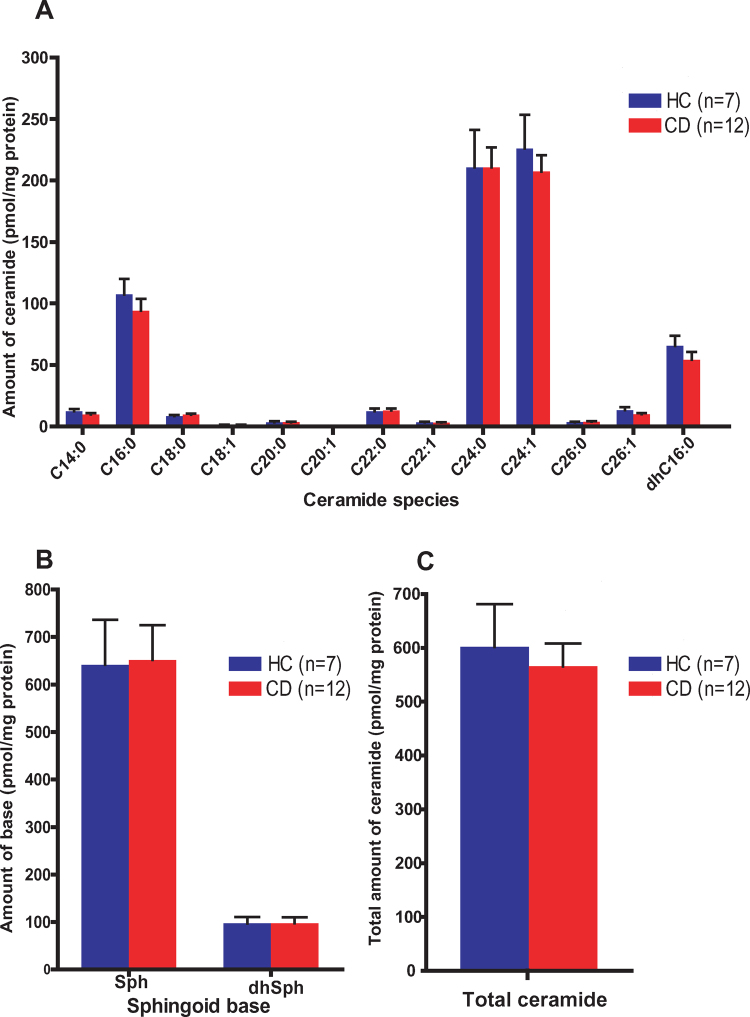
Ceramide and sphingoid base composition of HkEc-stimulated HC and CD macrophages. (A) Amounts of individual ceramide species expressed as pmol/mg protein. (B) Amounts of sphingosine (Sph) and dihydrosphingosine (dhSph). (C) Total amount of ceramide. No significant differences were found between HC (*n* = 7) and CD (*n* = 12) macrophages. Results are presented as mean + SEM.

Stimulation of HC macrophages with HkEc for 4 h resulted in a significant reduction in C16:0 (*p* < 0.05), C24:0 (*p* < 0.05) and C24:1 (*p* < 0.01) ceramide species, and a significant increase in dihydrosphingosine (*p* < 0.01) (Supplementary Fig. 1). Similarly, stimulation of CD macrophages with HkEc resulted in a significant reduction in C24:0 (*p* < 0.05) and C24:1 (*p* < 0.001) ceramides, with a concomitant increase in dihydrosphingosine content (*p* < 0.05) (Supplementary Fig. 1). There were no significant differences in the mean amounts of individual ceramide and dihydroceramide species (Supplementary Fig. 2A), sphingoid bases (Supplementary Fig. 2B), and total ceramide content (Supplementary Fig. 2C) between HC and CD macrophages in the HkEc stimulated state.

### 3.2 Macrophage phosphatidylcholine composition and dynamics alter following stimulation with *E. coli*, but are unchanged in Crohn's disease

**Supplementary Fig. 3:**
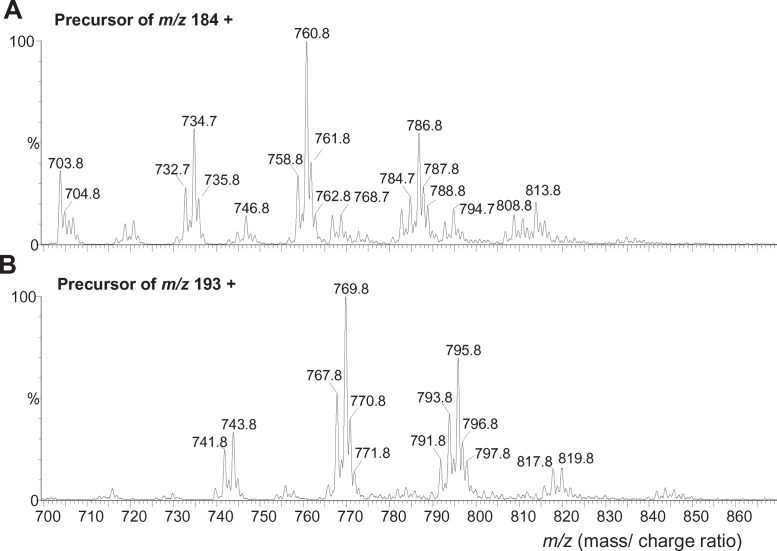
ESI-MS analysis of endogenous and newly synthesised PC. (A) Precursor scan of *m*/*z* 184+, indicating endogenous PC species. (B) Precursor scan of *m*/*z* 193+ showing newly synthesised PC species 9 *m*/*z* units higher than the endogenous species.

Endogenous and newly synthesised PC species over a 3 h time period were determined from precursor scans of *m*/*z* 184+ and *m*/*z* 193+ respectively. Representative PC spectra generated as precursor scans of *m*/*z* 184+ and 193+ are shown (Supplementary Fig. 3A and B). The predominant peaks at *m*/*z* 760.8 and 786.8 correspond to endogenous PC 16:0/18:1 and PC 18:0/18:2 (or PC 18:1/18:1) species respectively. PC species newly synthesised over the three hour time period can clearly be distinguished from endogenous PC, using the *m*/*z* 193+ precursor scan. The peaks at *m*/*z* 769.8 and *m*/*z* 795.8 correspond to newly synthesised PC 16:0/18:1 and PC 18:0/18:2 species respectively.

**Fig. 2:**
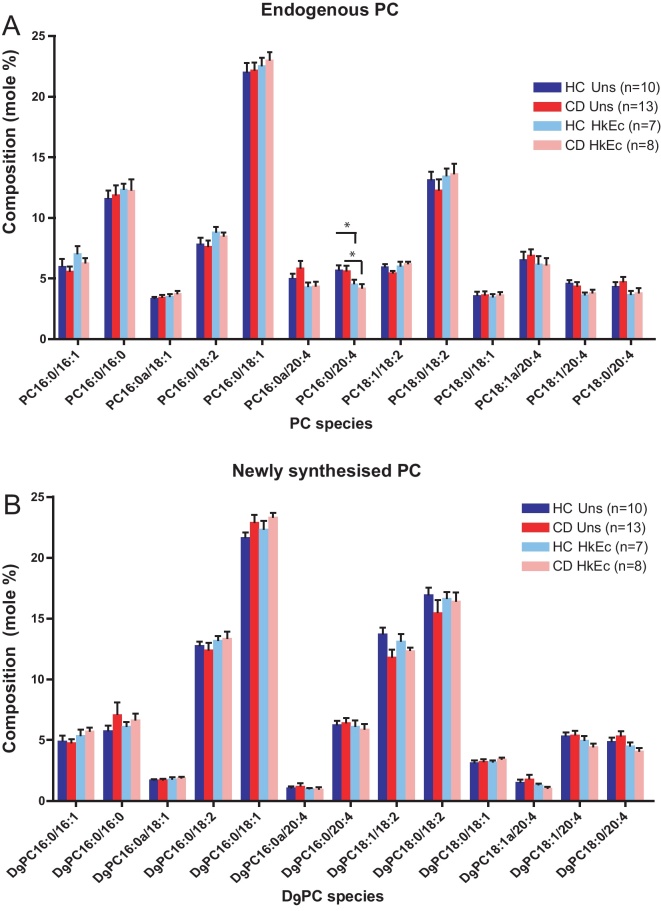
Composition of endogenous and newly synthesised PC in HC and CD macrophages, in the presence and absence of HkEc. Composition of (A) endogenous PC, consisting of various carbon chain length fatty acid species, and (B) newly synthesised (D_9_) PC in unstimulated (HC *n* = 10, CD *n* = 13) and HkEc-stimulated (HC *n* = 7, CD *n* = 8) macrophages, expressed as a molar percentage of total PC. Results are mean + SEM. * indicates *p* < 0.05.

In unstimulated HC (*n* = 10) and CD (*n* = 13) macrophages, the predominant endogenous PC species were PC 16:0/16:0, PC 16:0/18:1 and PC 18:0/18:2 (Fig. 2A). There were no significant differences in the molar percentage of any endogenous PC species between HC and CD macrophages. Stimulation with HkEc caused a reduction in the molar percentage of endogenous PC 16:0/20:4 in HC (*p* < 0.05) and CD (*p* < 0.05) macrophages. No significant differences were identified between CD and HC macrophages.

The profile of newly synthesised PC species was similar to that of the endogenous PC profile of HC and CD macrophages. PC 16:0/18:1 and PC16:0/18:2 were the predominant newly-synthesised species in HC and CD macrophages (Fig. 2 B). Stimulation with HkEc increased the proportion of PC 16:0/18:1 synthesised in HC macrophages, and increased the fraction of synthesised PC 16:0/16:1 in CD cells. There were no significant differences in the molar percentage of any species between HC and CD macrophages, either in the unstimulated state or after stimulation with HkEc.

**Supplementary Fig. 4:**
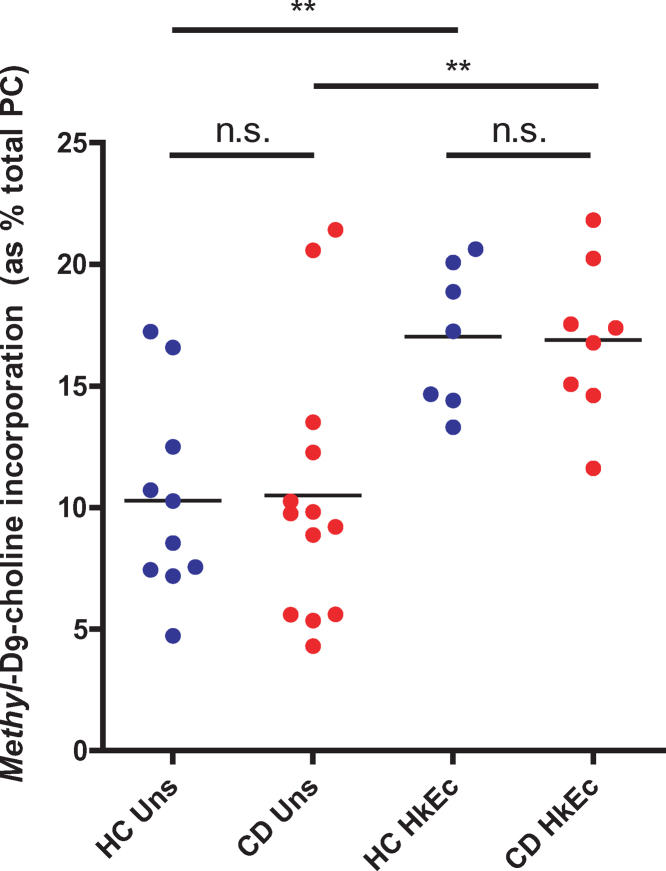
Incorporation of *methyl*-D_9_-choline into HC and CD macrophage PC. Total lipid was extracted and analysed by ESI-MS as precursor scans of *m*/*z* 184+ and *m*/*z* 193+. The fractional incorporation of *methyl*-D_9_-choline as a % of the total PC was determined.

The incorporation of *methyl*-D_9_-choline into PC was also determined over 3 h in HC and CD macrophages, as a measure of the global rate of synthesis of all PC species (Supplementary Fig. 4). The mean fractional incorporation of *methyl*-D_9_-choline into PC, thus rates of PC synthesis, were equivalent between HC and CD cells. Stimulation with HkEc increased the mean fractional incorporation of *methyl*-D_9_-choline into PC in HC macrophages (*p* < 0.01), and CD macrophages (*p* < 0.01) compared to unstimulated cells. Stimulation was therefore associated with an increased rate of PC synthesis; although no significant differences were observed between HC and CD macrophages.

### 3.3 Phosphatidylserine composition and dynamics do not differ between HC and CD macrophages

**Supplementary Fig. 5:**
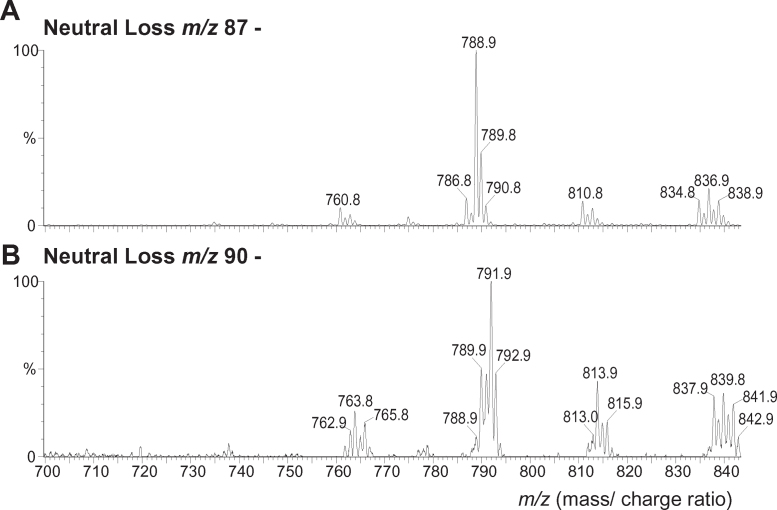
ESI-MS analysis of endogenous and newly synthesised PS. (A) Neutral loss scan of *m*/*z* 87 endogenous PS species. (B) Neutral loss scan of *m*/*z* 90, showing newly synthesised PS species 3 *m*/*z* units higher than the endogenous species.

**Fig. 3:**
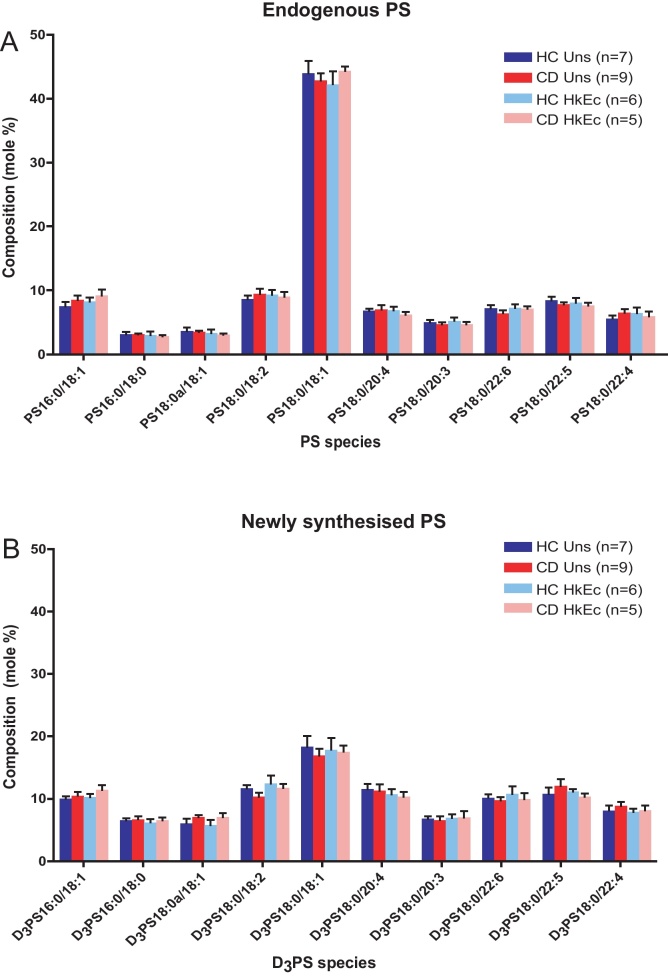
Composition of endogenous and newly synthesised PS in HC and CD macrophages, in the presence and absence of HkEc. Composition of (A) endogenous PS and (B) synthesised (D_3_) PS, in unstimulated (HC *n* = 7, CD *n* = 9) and HkEc-stimulated (HC *n* = 6, CD *n* = 5) macrophages. Results are expressed as a molar percentage of total PS and are mean + SEM. No statistically significant differences were identified between HC and CD.

Neutral loss scans of *m*/*z* 87 and *m*/*z* 90 were used to determine the profile of endogenous and newly synthesised PS species respectively in HC and CD macrophages. A representative neutral loss scan of *m*/*z* 87- is shown (Supplementary Fig. 5A); the peak at *m*/*z* 788.9 corresponding to the predominant endogenous PS species (18:0/18:1). A representative neutral loss scan of *m*/*z* 90- is also shown (Supplementary Fig. 5B); peaks at *m*/*z* 763.8, 789.9, 791.9 and 813.9 correspond to the predominant newly synthesised species PS 16:0/18:1, PS 18:0/18:2, PS 18:0/18:1 and PS 18:0/20:4. There were no significant differences in the molar percentage of any PS species between unstimulated HC and CD macrophages (Fig. 3A). There were no alterations in any endogenous PS species after stimulation with HkEc, and no significant differences between HC and CD macrophages in the stimulated state.

**Supplementary Fig. 6:**
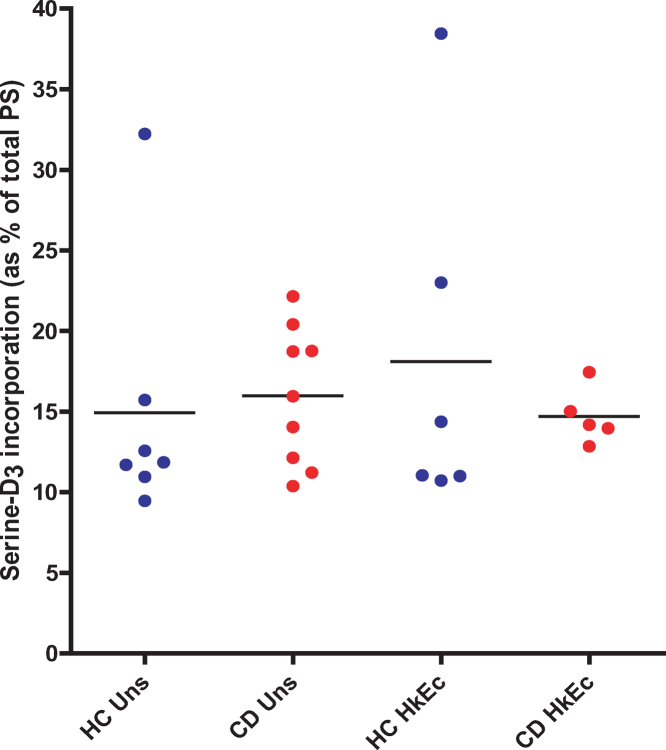
Incorporation of serine-D_3_ into HC and CD macrophage PS. Results are shown for unstimulated (HC *n* = 7, CD *n* = 9) and HkEc-stimulated (HC *n* = 6, CD *n* = 5) macrophages. Total lipid was extracted and analysed by ESI-MS as neutral loss scans of *m*/*z* 87- and *m*/*z* 90-. The fractional incorporation of serine-D_3_ as a percentage of the total PS was determined.

The predominant newly synthesised PS species over the 3 h time period in unstimulated HC and CD macrophages included PS 16:0/18:1, PS 18:0/18:1, PS 18:0/18:2 and PS 18:0/20:4 species (Fig. 3B). No significant differences were observed in the molar percentage composition of any PS species between unstimulated HC and CD macrophages. The profiles of the newly synthesised PS species were comparable between unstimulated and HkEc-stimulated macrophages, and similarly there were no differences in the molar percentage of any PS species between HC and CD macrophages. The rates of PS synthesis over 3 h were inferred from the fractional incorporation of serine-D_3_ within the total PS (Supplementary Fig. 6). There were no differences between HC and CD macrophages and, in contrast to PC, no alterations with HkEc stimulation.

### 3.4 Analysis of PI in HC and CD macrophages

**Supplementary Fig. 7:**
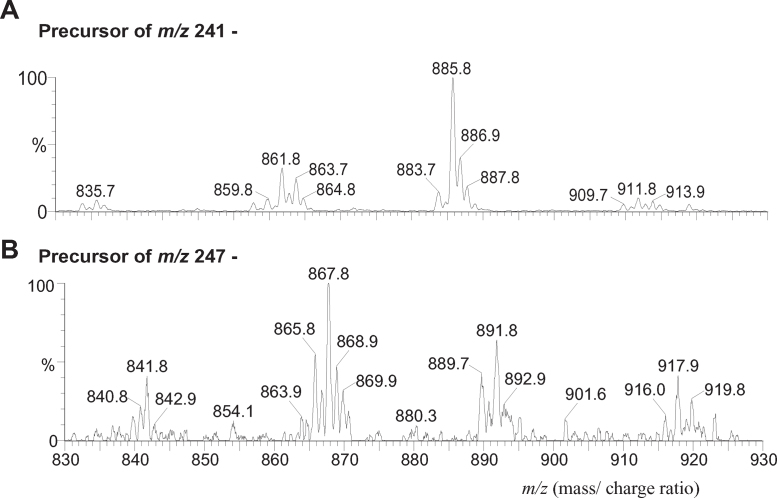
ESI-MS analysis of endogenous and newly synthesised PI species in macrophages. (A) Precursor scan of *m*/*z* 241-, indicating endogenous PI species. (B) Precursor scan of *m*/*z* 247-, indicating newly synthesised PI species.

**Fig. 4:**
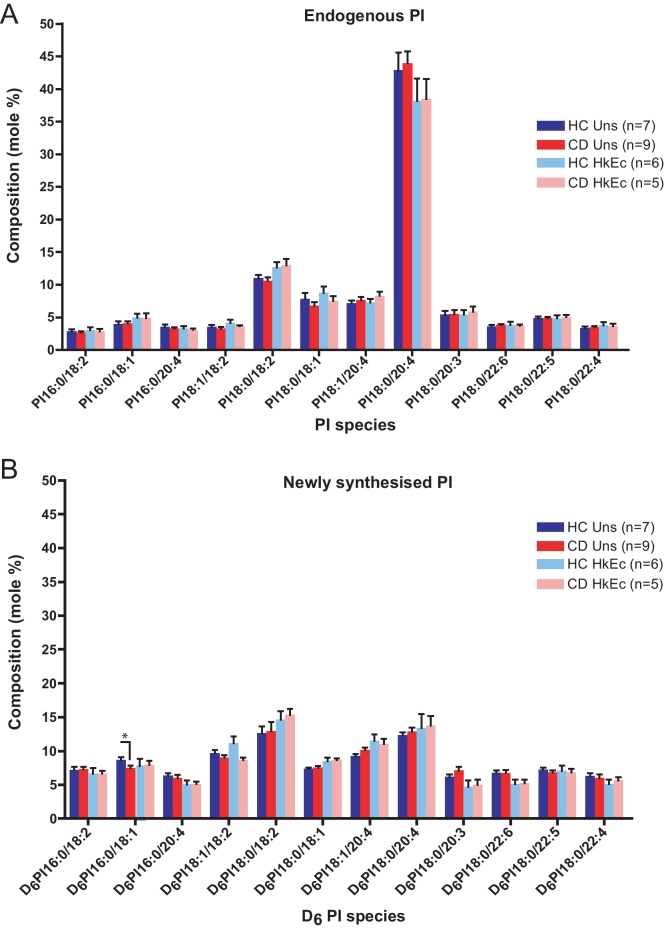
Composition of endogenous and newly synthesised PI in HC and CD macrophages, in the presence and absence of HkEc. Composition of (A) endogenous PI and (B) newly synthesised PI in unstimulated (HC *n* = 7, CD *n* = 9) and HkEc-stimulated (HC *n* = 6, CD *n* = 5) macrophages. Results are expressed as a molar percentage of the total PI and are mean + SEM. * indicates *p* < 0.05.

The composition of endogenous and newly synthesised PI species was determined by precursor scans of *m*/*z* 241- and *m*/*z* 247- respectively in HC and CD macrophages (Supplementary Fig. 7). The predominant endogenous PI species detected was PI 18:0/20:4, making up 42.9 ± 2.7% and 44.0 ± 1.8% of the total native PI in HC and CD macrophages respectively. The endogenous PI profiles were equivalent between HC and CD macrophages, and the composition was unaltered after HkEc stimulation (Fig. 4A).

The profile of newly synthesised PI species was strikingly different to that of endogenous PI, in both HC and CD macrophages (Fig. 4B). In contrast to endogenous PI, no single species predominated; PI 18:0/20:4 accounted for only 12.3 ± 1.1% of the newly synthesised PI in HC macrophages and 12.9 ± 1.8% in CD macrophages. 11 additional species accounted for the remaining newly synthesised PI. The proportion of newly synthesised PI 16:0/18:1 was significantly reduced in CD macrophages compared to HC in the unstimulated state (*p* < 0.05) (Fig. 4B).

**Supplementary Fig. 8:**
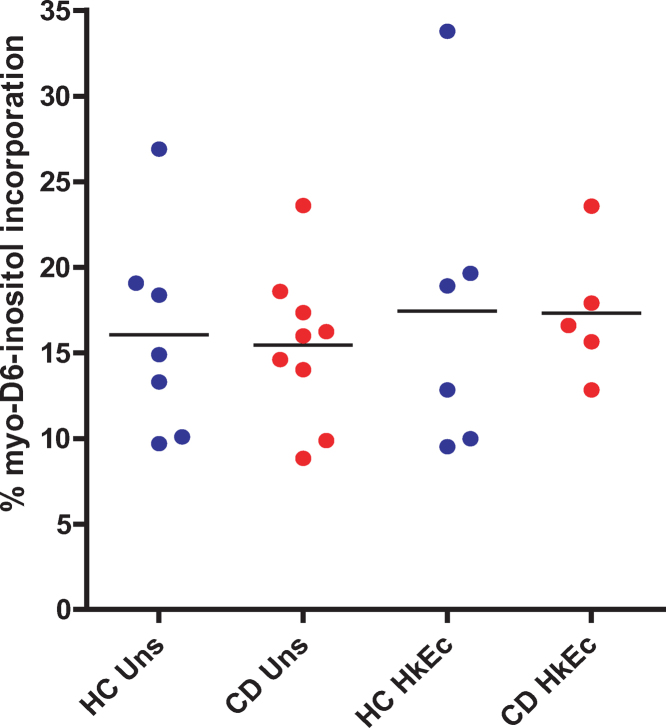
Incorporation of *myo*-D_6_-inositol into HC and CD macrophage PI. Results are shown for unstimulated (HC *n* = 7, CD *n* = 9) and HkEc-stimulated macrophages (HC *n* = 6, CD *n* = 5). No significant differences were identified.

The fractional incorporation of *myo*-D_6_-inositol into total cellular PI was calculated as a measure for the global rate of PI synthesis (Supplementary Fig. 8). The mean fractional incorporation of *myo*-D_6_-inositol as a percentage of the total PI was 16.1 ± 2.3% in HC and 15.5 ± 1.5% in CD macrophages over 3 h, indicating equivalent overall rates of PI synthesis in HC and CD. In contrast to PC, there was no alteration in the percentage incorporation after stimulation with HkEc, indicating comparable rates of PI synthesis in unstimulated and HkEc-stimulated macrophages.

### 3.5 Shotgun lipidomics analysis of ileal biopsies

**Fig. 5:**
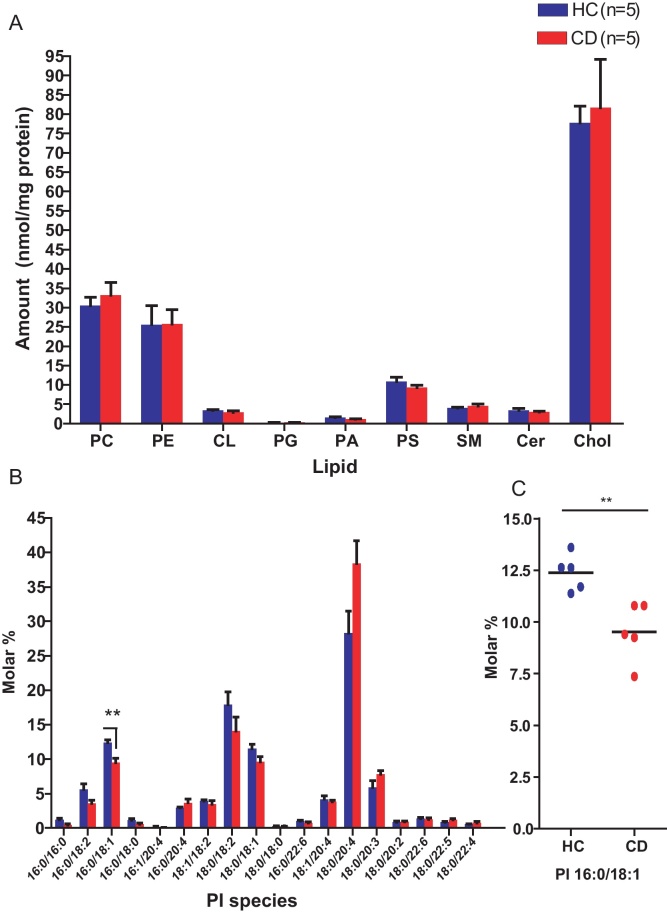
Shotgun lipidomics analysis of ileal biopsies. (A) Total phospholipid (PC, PE, CL, PG, PA, PS), sphingolipid (sphingomyelin, SM and ceramide, Cer) and cholesterol (Chol) content did not differ between HC (*n* = 5) and CD (*n* = 5) patients. (B) Molar percentage composition of phosphatidylinositol (PI) species. (C) Reduced molar percentage of PI 16:0/18:1 in CD biopsies compared to HC. ** represents *p* < 0.01.

Whilst the overall amounts of PC, PS, PG, phosphatidic acid (PA), cardiolipin (CL), sphingolipids and cholesterol in the biopsy samples did not differ between CD and HC (Fig. 5A), a significant reduction in PI 16:0/18:1 (as a percentage of total PI) was observed in CD compared to HC (*p* < 0.01) (Fig. 5B and C). This is the same species that showed reduced synthesis in CD macrophages compared to HC.

## 4 Discussion

No gross abnormalities were identified in the endogenous ceramide, dihydroceramide or sphingoid base composition of CD macrophages, both in the unstimulated state and after stimulation with HkEc. In addition, macrophage PC and PS composition, and rates of PC and PS and PI synthesis, were unaltered in CD macrophages; it is therefore unlikely that gross defects in these lipids underlie the impaired cytokine release observed in CD. Although previous studies have indicated differences in the fatty acid profiles of plasma phospholipids (Geerling et al., 1999) and PBMCs (Trebble et al., 2004) in CD patients, the inclusion of patients with active disease raises the possibility that the alterations observed could be a secondary phenomenon; indeed studies investigating quiescent patients have yielded conflicting results (Esteve-Comas et al., 1993). Secondly the specific profiles of macrophage ceramides, PC, PS and PI were determined in this study, whereas others have addressed fatty acid composition in terms of the total percentage of fatty acids or phospholipid species.

In CD macrophages, the relative percentage of newly synthesised PI 16:0/18:1 was reduced compared to HC, although this was not associated with an alteration in either the overall rates of PI synthesis or the molar percentage of endogenous PI 16:0/18:1. Importantly, shotgun lipidomic analysis of ileal biopsy samples also revealed abnormalities in this PI species in CD, suggesting that the observed reduction represents a genuine abnormality in CD. The suggestion of an altered profile of PI synthesis in CD patients is intriguing as minor variations in PI content and composition can exert major effects on the physical properties of membrane systems (Mulet et al., 2008). It is known that the p110δ isoform of PI 3-kinase, an enzyme involved in the generation of 3-phosphorylated phosphatidylinositol derivatives, is important in the TNF secretory pathway (Low et al., 2010). It is therefore possible that altered PI dynamics could contribute to the impairment in pro-inflammatory cytokine secretion observed in CD.

We have developed a three-stage model for CD, in which the first stage is failure of intestinal barrier function followed by impaired acute inflammation (Sewell et al., 2009). Phospholipids such as PI are important components of intestinal mucus as well as cellular components of the mucosa, and animal studies indicate a protective role for phospholipids in barrier function (Fabia et al., 1992). It is plausible that abnormalities in the mucosal PI profile could alter membrane or mucus fluidity, facilitating ingress of luminal contents into the bowel wall directly; alternatively, such alterations could enhance susceptibility to damaging emulsifiers such as bile acids. Although alterations in the phospholipid composition of intestinal mucus have previously been associated with ulcerative colitis rather than CD (Braun et al., 2009), PI composition was not determined in this study, which warrants further investigation.

The underlying mechanism of the reduced PI 16:0/18:1 observed in CD can only be speculated on at this stage. Given that the overall rate of PI synthesis is unchanged in CD macrophages, the difference could relate to an altered substrate preference of PI synthase in CD. Alternatively, abnormalities in the fatty acid metabolism pathway could lead to altered fatty acid availability for phospholipid synthesis. Notably, the recent GWAS meta-analysis identified a CD-associated SNP in a region containing the *FADS1* (Fatty acid desaturase 1) gene (Franke et al., 2010). This association adds credence to the hypothesis that fatty acid desaturation may be relevant in the pathogenesis of CD.

Stimulation of macrophages with HkEc was associated with alterations in ceramides and phospholipids, further implicating roles for these lipids in inflammation and innate immunity. LPS, TNF and IL-1β were previously shown to cause a rapid increase in the levels of ceramide in macrophage cell lines (MacKichan and DeFranco, 1999). In contrast with this previous work, HkEc stimulation in the present study was associated with a reduction in the C16:0, C24:0 and C24:1 ceramides, and a concomitant increase in dihydrosphingosine content. This difference could relate to differential effects of specific Toll-like receptor and HkEc stimulation on the sphingolipid pathway, or the different time frames investigated in the two studies.

Alterations in the phospholipid profile of macrophages observed after stimulation with HkEc included a reduction in the amount of endogenous PC 16:0/20:4. Recent application of lipidomic technologies to the activation of murine macrophages has demonstrated rapid generation of a wide range of eicosanoids and other oxylipins following mobilisation of arachidonate from major membrane phospholipids, including PC and PS (Rouzer et al., 2007). The decreased content of PC 16:0/20:4 in both control and CD macrophages after *E. coli* activation is consistent with such arachidonate mobilisation. Intriguingly, the fractional synthesis of PC 16:0/20:4 from D_9_-choline was unchanged in these activated cells, even though the rate of total PC synthesis was increased, implying there was no apparent deficit of arachidonate availability for PC synthesis under these conditions.

Comparison of patterns of D_9_-choline incorporation into PC with the profile of endogenous PC composition indicated a degree of co-ordinated acyl remodelling, with enhanced initial synthesis of all three 18:2-containing species. Endogenous PC was relatively enriched in disaturated and ether-linked PC species, which were presumably all formed subsequently to initial PC synthesis de novo by a variety of acyl exchange mechanisms. The extent of such acyl remodelling was even more apparent for PS and PI synthesis. The single species PS 18:0/18:1 accounted for over 40% of the total PS, but contributed less than 20% of PS synthesised from serine-D_3_. PS is formed by headgroup exchange from either PC or PE by the action of PS synthase (Hermansson et al., 2011), but acyl exchange mechanisms in PS synthesis have not been previously identified. Similarly, PI 18:0/20:4 was >40% of total PI, but only 12% of PI synthesis from *myo*-D_6_-inositol, substantiating previous suggestions that the high content of arachidonate in macrophage PI is maintained not by its synthesis de novo from *myo*-inositol but by direct acyl incorporation due to sequential activities of PLA_2_ and acyltransferases enzymes, proposed from incorporation patterns of D_8_-arachidonate (Balgoma et al., 2008).

Overall, in spite of the previous differences in cytokine release and intestinal barrier function, no major differences in lipid composition and synthesis were observed between tissue samples from CD patients and healthy controls, and their responses to bacterial stimulation. In contrast to studies of elicited bone marrow or peritoneal macrophages, all samples in our study were from individual subjects, and inter-subject differences in lipid nutrition could contribute to the variation in observed lipid responses. Nevertheless, the results question the extent to which lipid mobilisation is obligatorily linked to macrophage activation, and suggest a subtle difference in phosphatidylinositol composition in CD which could have an important influence on cytokine release and mucosal barrier function.

## Author contribution

Macrophage experiments were conceived and designed by GWS, AWS and ADP. GWS, JB, APW and YH conducted sphingolipid experiments. Phospholipid experiments were conducted by GWS, PJS, GK, VG and ADP. The shotgun lipidomics studies were planned by AWS and XH. FZR, RV and SLB contributed materials to this study. Data were analysed by GS, XH, ADP, GK and JB. GS, ADP and AWS wrote the paper.

## Figures and Tables

**Table 1:** Demographics of patients. Demographics of patients and healthy controls (HC) included in (A) sphingolipid study, (B) phospholipid studies and (C) shotgun lipidomics study.

A				
	Unstimulated		HkEc stimulated	
	HC	CD	HC	CD
Number	7	8	7	12
M:F	5:2	4:4	5:2	6:6
Mean age	44.1	36.2	44.1	39.7
Age standard deviation	15.6	12.9	15.6	14.8
Age range	23–63	19–63	23–63	19–65
Smokers	0	0	0	1
Treatment				
No medication		2		4
5-ASA		6		8

B				
	Unstimulated		HkEc stimulated	
	HC	CD	HC	CD
Number	10	13	7	8
M:F	5:5	6:7	5:2	4:4
Mean age	33.5	36.5	34.8	35.1
Age standard deviation	9.2	13.7	10.4	11.6
Age range	22–55	23–70	22–55	23–61
Smokers	1	1	0	1
Treatment				
No medication		6		3
5-ASA		7		5
Mean BMI	23.7	23.3	23.3	23.3
BMI standard deviation	3.5	3.0	3.8	3.7
BMI range	17.8–30.5	19.4–29.4	17.8–30.5	19.4–29.4

C		
	HC	CD
Number	5	5
M:F	1:4	3:2
Mean age	40.0	33.1
Age standard deviation	17.8	10.1
Age range	21–54	20–46
Smokers	0	0
Treatment		
No medication		3
5-ASA		1
Methotrexate		1
Previous resection		2
Active disease		2
